# Classification of Microcalcification Clusters in Digital Mammograms Using a Stack Generalization Based Classifier †

**DOI:** 10.3390/jimaging5090076

**Published:** 2019-09-12

**Authors:** Nashid Alam, Erika R. E. Denton, Reyer Zwiggelaar

**Affiliations:** 1 Department of Computer Science, Aberystwyth University, Aberystwyth SY23 3DB, UK; rrz@aber.ac.uk; 2 Norfolk and Norwich University Hospital, Norwich NR4 7UY, UK; erika.denton@nnuh.nhs.uk

**Keywords:** digital mammogram, microcalcification, stack generalization, classification, morphological features

## Abstract

This paper presents a machine learning based approach for the discrimination of malignant and benign microcalcification (MC) clusters in digital mammograms. A series of morphological operations was carried out to facilitate the feature extraction from segmented microcalcification. A combination of morphological, texture, and distribution features from individual MC components and MC clusters were extracted and a correlation-based feature selection technique was used. The clinical relevance of the selected features is discussed. The proposed method was evaluated using three different databases: Optimam Mammography Image Database (OMI-DB), Digital Database for Screening Mammography (DDSM), and Mammographic Image Analysis Society (MIAS) database. The best classification accuracy (95.00±0.57%) was achieved for OPTIMAM using a stack generalization classifier with 10-fold cross validation obtaining an A${}_{z}$ value equal to 0.97±0.01.

## 1. Introduction

**Figure 1:**
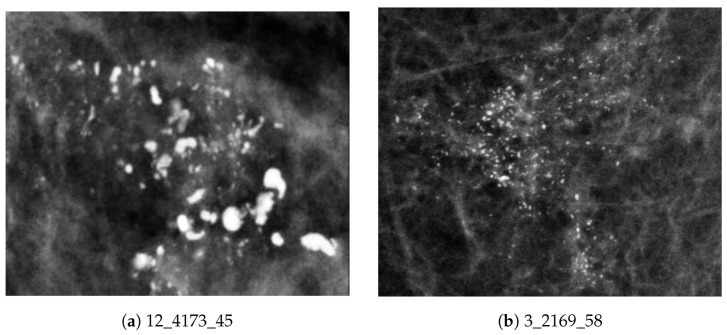
Example MC clusters from the OMI-DB database: (**a**) benign MC cluster; and (**b**) malignant MC cluster.

Breast cancer is one of the leading causes of cancer death in women [1,2]. The mortality rate of breast cancer can be reduced by early detection and by using Computer Aided Diagnostic (CADx) systems [3]. Microcalcification (MC) clusters are an important early sign of breast cancer [4]. MC clusters appear as small localized granular points of high brightness within soft breast tissue [5] and it can be difficult to distinguish MC clusters from normal breast tissue because of their subtle appearance and ambiguous margins [6,7]. Approximately 50% of early diagnosed cases indicate the existence of MC clusters, revealing up to 90% of ductal carcinoma in situ [8]. Typical examples of benign (non-cancerous) and malignant (cancerous) MC clusters are shown in Figure 1.

Double reading can improve sensitivity, but a lack of experienced radiologists can be a challenge [9]. CADx can assist radiologists in detecting abnormalities in an efficient way [10,11] and systems have been developed to provide a second opinion for diagnosis [12]. Previous studies have developed computerized methods to aid the diagnosis of MC clusters. Singh et al. [13] proposed a MC cluster classification technique based on morphology: including size of the calcifications and number of calcifications in a cluster. A region of interest (ROI) around the MC cluster was first enhanced using morphological operations, and two types of features, namely cluster shape and cluster texture, were obtained. A new set of shape features generated by recursive subsampling was added to the feature set, which improved the classification accuracy. Akram et al. [14] proposed an improved Fisher Linear Discriminant Analysis (LDA) approach for the linear transformation of segmented micro-calcification data. In the proposed method, a SVM variant was used to classify benign and malignant clusters. Multi-scale graph topological features were used by Chen et al. [15] using a k-nearest-neighbors classifier. The performance of machine learning techniques was investigated by Rampun et al. [16] by examining the probability outputs from classifiers in conjugation with the classification accuracy and area under the receiver operator curve (A${}_{z}$) to indicate the reliability of CADx.

Bekker et al. [17] proposed a two-phase classification scheme. The method was based on combining decisions from multiple views (craniocaudal (CC) view and mediolateral oblique (MLO) view), implemented by a logistic regression classifier, followed by a stochastic combination of the two view-level (CC and MLO) indications into a final benign or malignant decision. Shachor et al. [18] examined data fusion methods for multi-view MC cluster classification. This data fusion concept was implemented by a special purpose neural network architecture that demonstrated the task of classifying breast microcalcifications as benign or malignant based on CC and MLO mammographic views.

Hu et al. [19] applied a hidden Markov tree model of dual-tree complex wavelet transform (DTCWT-HMT) for microcalcification diagnosis in digital mammograms. DTCWT-HMT was used to capture the correlation between different wavelet coefficients and model the statistical dependencies and non-Gaussian statistics of real signals. The combined features of the DTCWT-HMT and the DTCWT were optimized by a genetic algorithm (GA). An extreme learning machine (ELM) was used as the classifier to diagnose the benign and malignant MC clusters.

A feature selection method was introduced by Diamant et al. [20] based on a mutual information (MI) criterion for automatic classification of MC clusters. The MI based feature selection method was explored for various texture features. Wang et al. [21] used a semi-automated segmentation method to characterize all MCs, and constructed a classifier model to assess the accuracies for microcalcifications and breast masses, either in isolation or in combination, for classifying breast lesions. Sert et al. [22], however, used convolutional neural networks along with various preprocessing techniques such as contrast scaling, dilation, cropping, etc. to classify microcalcification. Adaptive thresholding and morphological technique was used by Nguyen et al. [23] to segment nuclei for single channel image. A superpixel-based framework was presented for segmentation that used a “hybrid” approach which was intended to integrate the advantage of region-based clustering algorithm and an edge detector with an integrated edge map.

**Figure 2:**
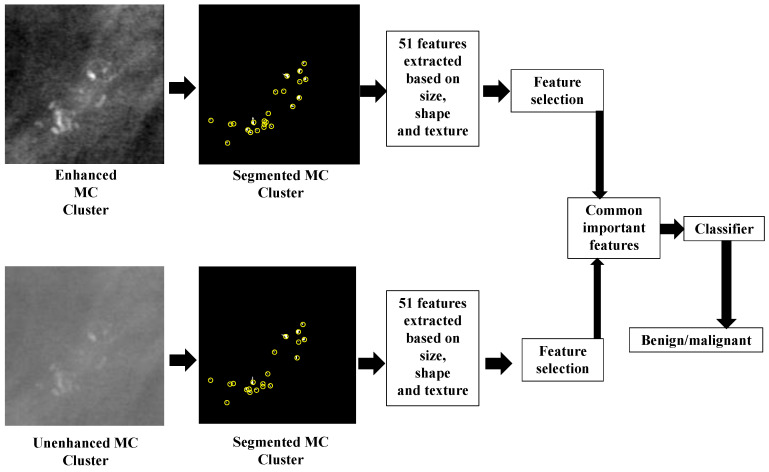
Overview of the proposed MC cluster classification methodology.

The present work focused on developing a method for discriminating malignant and benign clusters in digital mammograms. Images were first segmented using a wavelet-based method in conjunction with a bi-cubic interpolation technique and a series of morphological operations. A combination of morphological, texture, and distribution features from individual MC components and the MC cluster were extracted and MC clusters were classified with a stack generalization-based classifier. An ensemble classifier was also used to classify MC clusters from digital and digitized mammograms. The most important features were selected and used to classify the MC cluster as benign or malignant. An overview of our proposed approach is presented in Figure 2.

## 2. Materials and Methods

### 2.1. Image Databases

We used the digital mammograms from the Optimam Mammography Image Database (OMI-DB) [24], which is currently an ongoing project at the Medical Physics Department of the Royal Surrey County Hospital, which contains NHS Breast Screening Programme (NHSBSP) images from different centres across the United Kingdom with an aim to develop a large repository of breast images for research purposes. The database contains 3D and 2D unprocessed and processed breast images, associated annotations and where applicable expert-determined ground truths, which describe features of abnormalities such as microcalcification, mass, architectural distortions, etc. The images were categorized by radiologists into three clinical categories: normal, benign, and malignant. Core biopsies were also performed where applicable and associated with the opinion provided by the radiologists. In our experiment, patient-based case selection was performed on the digital mammograms, and a total number of 286 cases (136 benign and 150 malignant) were selected, which only contained microcalcification clusters that had associated core biopsy scores. The histological and radiographic scores were not considered for patient-based case selection, as very few images that contained microcalcification clusters were provided with such scores, which was an obstacle to create a balanced database. These mammograms were acquired using a Hologic Selenia mammography unit, with a resolution of 70 microns per pixel and a depth of 12 bits [25].

The evaluation also used the digitized mammograms from two different publicly available benchmark databases: the Mammography Image Analysis Society (MIAS) [26], and the Digital Database for Screening Mammography (DDSM) [27]. The DDSM database contains cranial-caudal (CC) and mediolateral oblique (MLO) views of left and right breasts of each patient. The images containing suspicious area have pixel-level “ground truth” information of the abnormality, and a malignancy assessment on a five-point scale according to the American College of Radiology (ACR) Breast Imaging Reporting and Data System (BIRADS) [28]. In total, 280 digitized mammograms containing MC clusters (148 benign and 132 malignant) were used. The MC clusters colocated with masses were not considered, as the existence of mass could mislead the classification results whilst considering the neighborhood of MCs to extract relevant features. The cases were selected at a patient level, and only MLO views were used. The mammograms in the DDSM database were digitized by one of four different scanners: DBA M2100 ImageClear (42 microns per pixel, 16 bits), Howtek 960 (43.5 microns per pixel, 12 bits), Lumisys 200 Laser (50 microns per pixel, 12 bits), and Howtek MultiRad850 (43.5 microns per pixel, 12 bits). For our experiment, only the mammograms obtained using Lumisys 200 Laser scanners were considered to keep inline with the pixel size of another digitized database (MIAS) [26] used for the development and evaluation of the proposed system. The MIAS database [26] contains 322 images, among which 24 cases (12 benign and 12 malignant) contain microcalcification clusters. The mammograms in the MIAS were digitized to 50 microns per pixel. The truth-marking of the locations of the abnormalities were delineated by an expert radiologist.

### 2.2. Preprocessing and Segmentation

Enhancement was necessary as MC clusters are usually very small, and sometimes can be situated in dense breast tissue with very low visibility. This phenomenon makes the segmentation and classification task more challenging [11]. To overcome this problem, a wavelet-based algorithm was applied to enhance the mammograms, and the contrast between the MC cluster and surrounding background tissues was increased (see Section 2.2.1). Such contrast enhancement facilitated the subsequent MC cluster segmentation, as described in Section 2.2.2. Features of MC clusters were extracted from the segmented image and were used to classify the clusters as benign or malignant.

#### 2.2.1. Mammogram Enhancement and Patch Extraction

**Figure 3:**
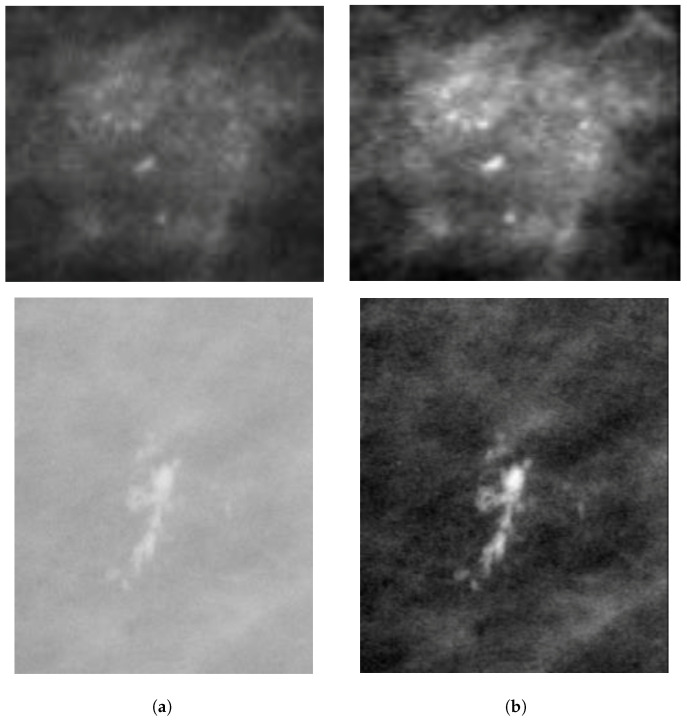
Example enhancement of MC clusters: digital mammogram from the OMI-DB database (top row: 1_1076_463) and digitized mammogram from the DDSM database (bottom row: B_3049_1.RIGHT_MLO): (**a**) MC patch cropped from the original mammogram (without image enhancement); and (**b**) MC patch cropped after enhancement.

A dynamic wavelet-based algorithm [29] was applied to enhance the mammograms. The Discrete Wavelet Transform (DWT)- based method was used because of its low computational complexity and special transformed domain properties [30]. The process of mammogram enhancement was divided into three parts, which included decomposition, sharpness estimation and filtering. The image was first decomposed into individual sub-bands using a multi-level separable DWT [31,32]. The log-energies of the vertical, horizontal, and diagonal sub-bands at each decomposition level were calculated followed by measuring the total log-energy (TLE) of each level. Subsequently, by combining the TLE of each decomposition level [29], the Scalar Sharpness Index (SSI) was calculated. The SSI was later used to estimate the overall sharpness of the images. Higher values of SSI were considered as an indicator of higher sharpness of the image. More details on the wavelet-based enhancement algorithm were described by Misra et al. [29], who applied the enhancement approach to satellite images. To enhance the mammograms, the number of sub-bands and the image decomposition level were chosen as 3, as we aimed to obtain the horizontal, vertical and diagonal details from the mammograms. Each sub-band was assigned a predefined weight (0.10) to enhance the diagonal higher spatial frequency. The weight was set to 0.10, as an increase in weight above 0.8 did not provide further increase in enhancement and a weight less than 0.8 provided decay in enhancement. The region containing the MC cluster was cropped (see Figure 3b) from the enhanced mammogram using the provided annotations. The effect of the enhancement algorithm is shown in Figure 3b, where it can be noted that the appearance of MC clusters is enhanced for both digital (OMI-DB) and digitized (DDSM) mammograms from a qualitative point of view.

#### 2.2.2. Probability Image Generation for MC Cluster

**Figure 4:**
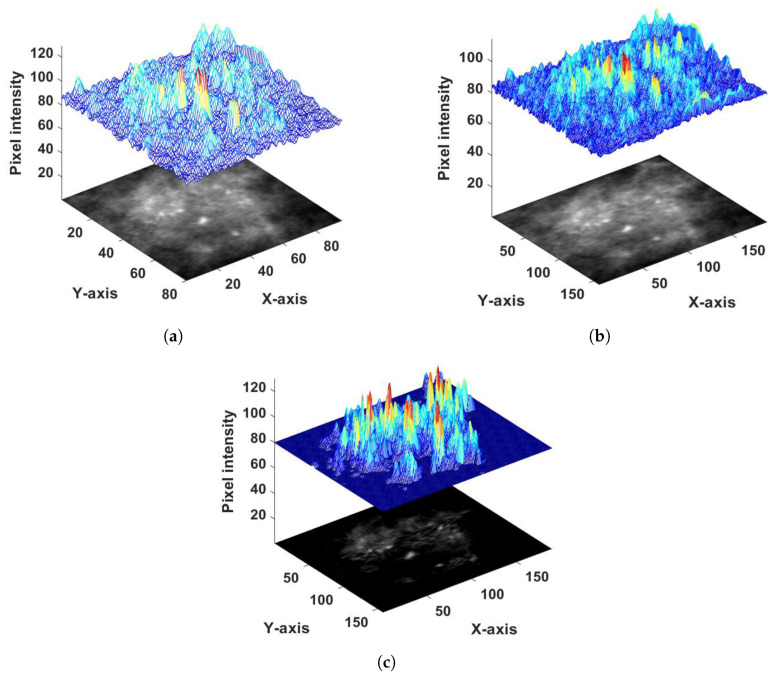
(**a**) Three-dimensional intensity representation of a 158×189 pixel area of a digital mammogram; (**b**) calculated object background intensity of the same area; and (**c**) the difference image between the original image (**a**) and the background image (**b**).

A combination of image interpolation, morphological operations, and edge-preserving filtering was applied to generate the probability image of the MC clusters. The enhanced cropped region of interest (ROI), containing the MC cluster, was considered as a three-dimensional plot with the *z*-axis representing the intensity of each pixel (see Figure 4a). The whole image was first divided into 30×30 sub-regions. The size of sub-regions was set to 30×30 to maintain a trade-off between over-segmentation and under-segmentation of the MC clusters. Choosing sub-regions bigger than 30×30 would result in over-segmentation in low contrast images where the disparity between the MC cluster and their background is very low. Choosing a size less than 30×30 would cause under-segmentation.

**Figure 5:**
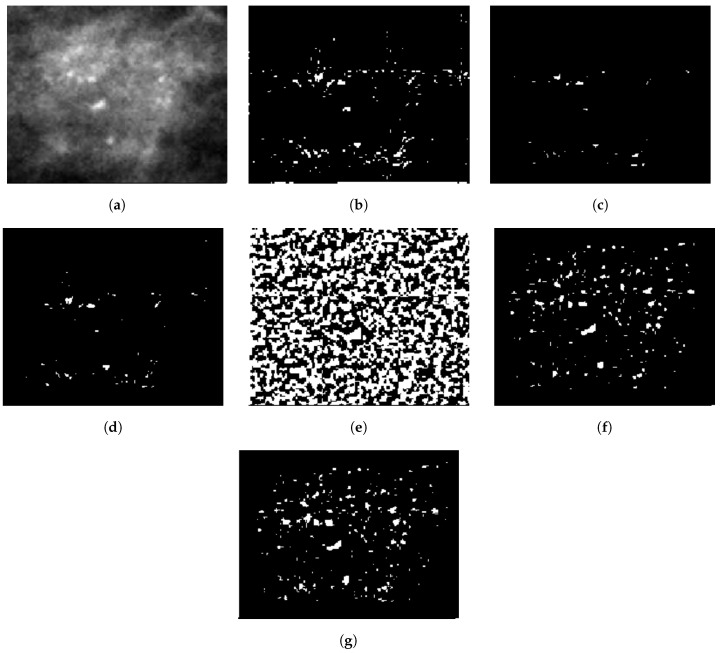
(**a**) Enhanced image patch (1_1076_463) from the OMI-DB database; (**b**) binary image containing 5% of the highest positive intensity values from the difference image; (**c**) eliminating single pixels and perform erosion on (**b**); (**d**) Image A: pixels having higher value than the specified threshold mention in Section 2.2.2 are added to (**c**); (**e**) contrast enhancement filter applied to the bi-cubic interpolated image of (**a**); (**f**) Image B: five percent of the pixels having the highest intensity are selected from the filtered image; and (**g**) Image C: Logical summation of (**d**) and (**f**).

Bi-cubic interpolation [33] was applied to each sub-region to obtain pixel intensities of the background tissue (see Figure 4b). The resulting image (Figure 4b) was subtracted from the original image (Figure 4a) to obtain the difference between the original and local background pixel values (Figure 4c). In Figure 4b,c, high picks indicate higher pixel intensities and sharp edges in the image. From this difference image (Figure 4c), the pixels with positive values were identified and a percentage of these (5%) with the highest values was selected to generate a binary image (see Figure 5b). The reason for selecting the 5% highest pixel values was to avoid under-segmentation. The highest positive pixel values considered as MC clusters were characterized by higher intensity compared to their local background tissue. Single pixels were removed from the generated binary image, and an erosion operation was performed to eliminate false positive pixels (see Figure 5c). To perform the erosion operation, a square structuring element of size 3×3 was used with all values set to one to retain the original morphology of the segmented MC cluster. The lowest value among the 5% selected pixels was specified as a threshold. If the number of the existing pixels, in Figure 5c, was lower than 10% of the total number of pixels in the cropped image patch, the pixels with intensity higher than half of the previously specified threshold were included in the binary image (see Figure 5d). Overall, 10% of the total pixels in the cropped image patch maintained a trade-off between over-segmentation and under-segmentation. By doing so, enough pixels were generated for the binary image (A) (see Figure 5d). The above procedure was performed to avoid under-segmentation when the mammogram exhibited very low contrast, which was usually due to erroneous exposure  conditions.

Subsequently, a contrast enhancement filter, having a 9×9 kernel with its central pixel element equal to 80, was applied to the bi-cubic interpolated image [33] (see Figure 5e). Five percent of the pixels having the highest intensity were selected from the filtered image, producing another binary image (B) (see Figure 5f). Finally, logical summation (AND) of the two binary Images A and B (Figure 5e,f) was performed to keep pixels that have high intensity values in comparison with the background intensity of their local neighborhood tissues (see Figure 5g).

#### 2.2.3. Specifying MC Cluster

The clinical definition of the MC cluster was used for the reduction of false positives from the probability image generated in Section 2.2.2. According to the medical definition of clustered MC, more than three MCs should reside in a 1 cm${}^{2}$ area [34], which is equivalent to 200×200 pixels in the digitized data (DDSM and MIAS) with a pixel size equal to 50 μm, and 143×143 pixels in the digital data (OMI-DB) with a pixel size equal to 70 μm. This results in 143×143 pixel equivalent to 1 cm${}^{2}$ block area for OMI-DB, and 200×200 pixel equivalent to 1 cm${}^{2}$ block area for DDSM and MIAS.

**Figure 6:**
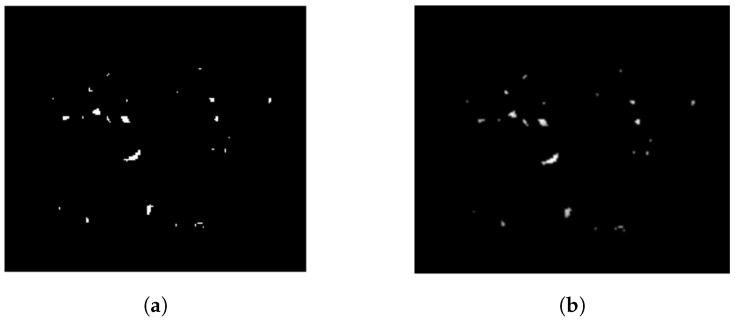
(**a**) Elimination of blobs containing one or two pixels from the probability image generated in Section 2.2.2 (see Figure 5g); and (**b**) final probability image, for example case (1_1076_463), after discarding all blobs from 1 cm${}^{2}$ pixel block whilst objects inside the block were less than 3. In this example, all the 1 cm${}^{2}$ pixel blocks contained more than three blobs so no object elimination was done.

From the probability image generated in Section 2.2.2 (see Figure 5g), regions containing one or two pixels were removed, as they were considered artifacts [35], and an erosion operation with a 2×2 unit element kernel was performed (see Figure 6a). Here, a 2×2 unit element kernel was used for the erosion operation, as a bigger kernel size generated under-segmented images and a smaller kernel had barely any effect. Removal of individual objects with a morphological erosion operation was necessary, because the diagnostic information was based on the existence of a group of MCs [34]. Subsequently, neighboring pixels with eight connectivity were grouped together [11] and, considering the clinical definition of MC cluster formation, the binary image having only eight-connected component was divided into 1 cm${}^{2}$ block areas. This results in 143×143 pixel equivalent to 1 cm${}^{2}$ block area for OMI-DB, and 200×200 pixel equivalent to 1 cm${}^{2}$ block area for DDSM and MIAS.

**Figure 7:**
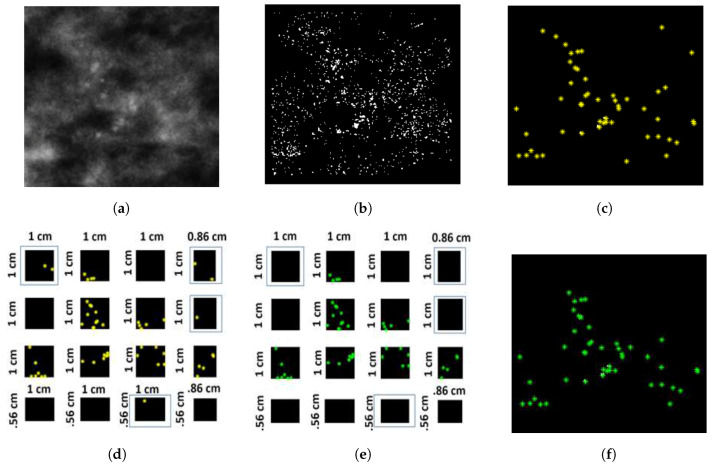
(**a**) Enhanced image patch (10_35_242) from the OMI-DB database; (**b**) Image C: logical summation of two binary Images A and B for image patch (10_35_242); (**c**) eliminating single pixel from (**b**) (all MCs are highlighted for better visual understanding); (**d**) dividing (**c**) into 1 cm${}^{2}$ pixel blocks: the blocks containing fewer than three MCs are marked by a rectangle, the last row and the last column of image blocks were not 1 cm${}^{2}$ pixel block as they were adjusted according to the patch image size; (**e**) elimination of all MCs inside each 1 cm${}^{2}$ pixel block that contained fewer than three MCs (marked by a rectangle); and (**f**) all blocks in (**e**) are stitched together to produce the final segmented image.

Elimination of all the elements inside each 1 cm${}^{2}$ block area were done; where the minimum number of objects inside a block was less than 3 [34], all the elements were removed (see Figure 6b). In Figure 6b, no object elimination was done inside any block since all the 1 cm${}^{2}$ blocks contained more than three objects; a sample case is shown in Figure 7, which represents how the images were divided into 1 cm${}^{2}$ block areas, and the elements inside each block were eliminated, where the minimum number of objects inside the block was less than 3 [34]. For better visual understanding, the MC clusters were highlighted in yellow (see Figure 7c,d) and green (see Figure 7e,f). Image C was generated for the sample image patch (10_35_242) from the OMI-DB database (Figure 7b). All single pixels were eliminated to remove a fraction of false positive MC objects (Figure 7c). The image was then divided into 1 cm${}^{2}$ pixel blocks (see Figure 7d). The blocks containing less than three MCs, marked by a rectangle, were removed (see Figure 7e). All blocks were stitched together to generate the final segmented image (Figure 7e).

The whole MC cluster may not be covered by the proposed approach. The block area has to be slid to different locations of the patch image to build up a complete MC cluster network. For the sliding window approach, we would have to come up with a methodology to harmonize the changes in MC clusters between windows and how this representation is affecting the classification. In addition, the sliding window approach would be time consuming, and is an interesting research question to address in future.

## 3. Segmentation Evaluation

**Figure 8:**
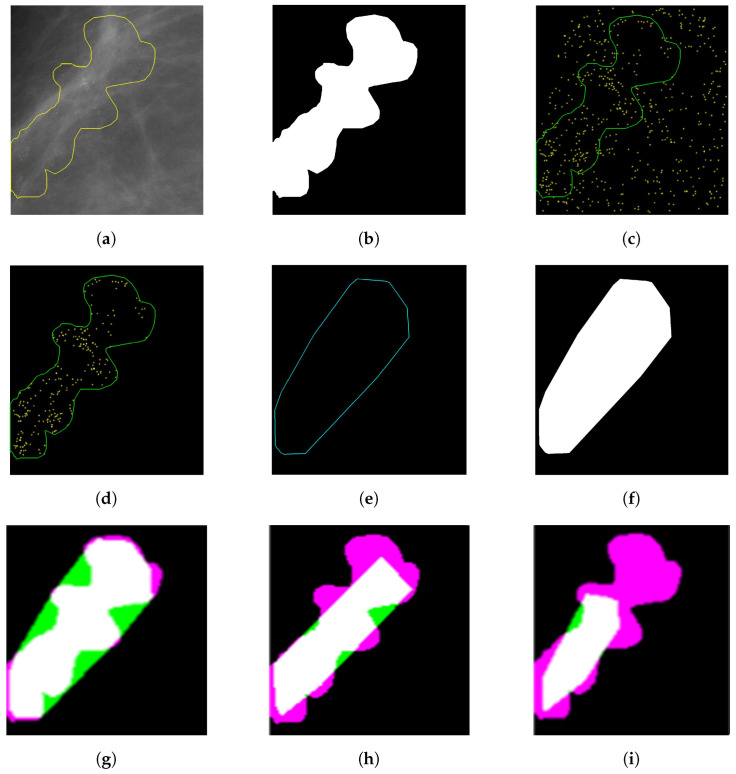
(**a**) Annotation by radiologist (B_3121_1.RIGHT_MLO); (**b**) reference MC cluster mask generated from (**a**); (**c**) border extraction from reference MC mask and overlaid on segmented image generated using morphological segmentation approach; (**d**) MC resides inside the border annotated by expert radiologist; (**e**) convex hull outline using the border points of segmented blobs residing inside annotation outline; (**f**) mask generation from convex hull border of segmented image; (**g**) Dice similarity score (based on morphological segmentation approach) = 0.85599 (white region, true positive; green region, false positive; magenta region, false negative); (**h**) Dice similarity score (based on Oliver’s [38] segmentation approach) = 0.76514; and (**i**) Dice similarity score (based on area ranking segmentation approach) = 0.5494.

**Figure 9:**
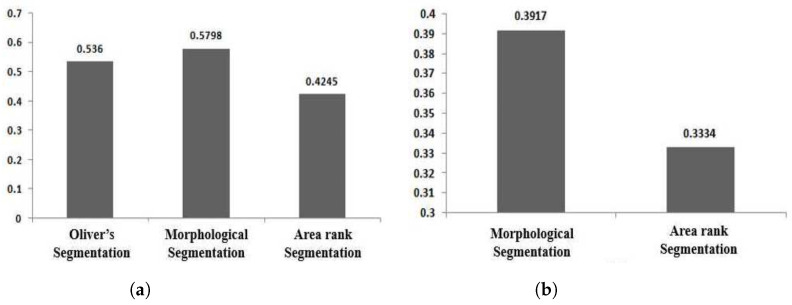
(**a**) Dice similarity score to compare segmentation results of Oliver’s segmentation method, and our proposed two segmentation methods using the DDSM database; and (**b**) Dice similarity score to compare segmentation results of our proposed two segmentation methods using the MIAS database.

The evaluation was carried out using the Dice similarity metric [36,37], and is in line with our previous work [11]. The reference masks (see Figure 8b) were generated from the radiologist’s annotation outline (see Figure 8a). Subsequently, individual MCs that reside inside the radiologist’s annotation were considered to generate convex hull. This convex hull (see Figure 8f) and the reference mask (see Figure 8b) were used to calculate the Dice similarity score (see (Figure 8g–i)). The Dice similarity metric for DDSM and MIAS is presented in Figure 9.

In Figure 9, it is clear that the segmentation technique based on the morphological approach works better than the area-rank based segmentation method proposed in [11]. In addition, it is worth noting that the segmentation results generated by applying the method of Oliver et al. [38] give almost the same similarity score as gained by our proposed morphological operation-based segmentation method, although the similarity score for our proposed approach is slightly higher than with Oliver’s method [38].

## 4. Classification Module Construction

To classify MC clusters into benign or malignant, a series of classification algorithms was explored to create an ensemble learner instead of using only one classification method. A set of nine different machine learning algorithms was used: k-nearest neighbor (kNN) classification [39], a multilayer perception (MLP) classifier [40], a classification tree [41], random forest [42], support vector machines using four different kernels (Gaussian RBF, sigmoid, linear, and polynomial) [43], and a Naive Bayes network [44]. All the classifiers individually provide a binary decision by classifying the images as benign or malignant. Each classification algorithm was separately applied to the images and the number of malignancy predictions (votes for malignancy) were counted. Afterwards, the total number of malignancy prediction was divided by the total votes. For example, if eight of the nine classifiers classified a case as malignant, then the final estimation of the ensemble classifier for malignancy would be 89%. The advantage of employing an ensemble classifier was to aggregate a set of models to provide more robust classification results rather than using the opinion from a single classification model. The predictions from individual classifiers were combined using majority voting, and as such the possibility of over-fitting of any particular classifier was avoided. The individual classification results from different classification algorithms are presented and discussed in Section 6.

A stacked generalization [45] approach was also applied to create a classifier for classifying the MC clusters. In this approach, the above-mentioned nine learning algorithms were considered as base classifiers, and the Naive Bayes classifier [44] was used as the meta-classifier (combiner), as a previous experiment [46] confirmed that the Naive Bayes classifier as a combiner performed better than majority voting. In a stacked generalization approach, the meta-learner was used instead of averaging to combine predictions of the base classifiers. Predictions of the base classifiers were used as input for the meta-classifier. The meta-classifier attempted to learn the relationships between predictions and the final decision. The meta-classifier also corrected some mistakes of the base classifiers [45].

The aim of this research was to investigate the merit of using a conventional stack generalization approach to classify MC cluster in mammogram. Using modern methods such as auto-encoders or generic neural networks for feature selection and classification is an interesting research question to be addressed in the future [47,48].

## 5. Feature Extraction and Feature Selection

It is crucial to extract and select appropriate features that can classify MC clusters into their clinical categories. MC clusters can be assessed based on specific properties such as: size, shape, number, distribution, etc. [33]. A set of 51 features [49] was computed from the segmented blobs (see Section 2.2.3) for extracting the statistical and morphological properties of the MC clusters, which form the feature space. All computed features characterize either an individual MC or an MC cluster. These features were grouped into three categories: shape, size and texture (see Table 3). Since the number of computed features was large and their discriminating power varied (see Table 3), a feature selection approach was used to obtain the most salient features. More details on the performance of individual features to classify MC clusters are discussed in Section 6.

Feature selection was done by employing the CfsSubsetEval [50] attribute evaluator and the BestFirst search method [51] in Weka [51]. CfsSubsetEval [50] evaluated the significance of a subset of features by approximating the individual predictive ability of each feature and the redundancy between them: this meant that features that were highly correlated with the class whilst having low inter-correlation were more likely to be selected [51]. On the other hand, BestFirst [51] searched the feature space subsets by greedy hill-climbing augmented with a backtracking facility [51], which could start from any point and search forwards and backwards, by considering all possible single feature vector additions and deletions [52]. The selected features from unenhanced images were put into a group (α). Subsequently, the same 51 features were extracted from the segmented images that were generated from the enhanced mammograms. The most significant features from the enhanced images were gathered into another group (β), using the same feature selection technique. The common features from group α and group β formed a new feature space.

To ensure the robustness of the feature selection and avoid bias, all data were divided using 10-fold cross-validation scheme and 9-fold cross-validation scheme, respectively. Important features were extracted using the images residing in each fold, which showed the same features extracted consistently. When the images were split into different number of groups by changing the fold-number higher and lower than 10, we constantly obtained the same set of features extracted. A similar approach was applied to measure the robustness of the feature selection in a previous publication [49].

The feature extraction and selection technique, as mentioned above, was applied separately on the digitized and digital databases to investigate whether the provided features from the digital database outperformed those extracted from the digitized database in classifying MC clusters. Table 1 represents the four most important features extracted and selected using Digitized database (DDSM), and Table 2 represents the two most important features extracted and selected using the Digital database (OMI-DB) with the associated clinical interpretations.

The in-depth details on the impact of our feature selection approach are described in Section 6. Here, all images were segmented maintaining the clinical grounding of the distribution of the MC cluster which indicate that an area of 1 cm${}^{2}$ contains no fewer than three MCs [34]. The spatial resolution of mammography is normally ranging from 40–100 µm per pixel, which enables detection of MC clusters at an early stage [15]. The aforementioned feature extraction and selection method was also employed on the segmented images from the digital and digitized databases by randomly considering a 100×100 pixel area as 1 cm${}^{2}$, to investigate if this had an impact on the MC cluster classification. The results are presented in Table 6 in Section 6.

To evaluate the reliability of the feature selection approach, images from the digital and digitized databases were separately divided into ten folds. The process of feature selection was performed on each fold, which indicated the same selection of features. Detailed evaluation of the feature selection for MC cluster classification is provided in Section 6.

## 6. Result Analysis

To investigate the influence of shape, size, and texture aspects, each individual feature type was separately used for the classification using ensemble learning (see Table 3). The experiment was separately applied on the individual databases, where the features cognate with size provided the highest A${}_{z}$ values over the shape and texture features for both digital and digitized databases with no feature selection. Whilst only considering the size features, the highest A${}_{z}$ value (0.87±0.01) was gained for the digital database (OMI-DB). With feature selection, as described in Section 5, the value of A${}_{z}$ was 0.83±0.01 for OMI-DB, 0.72±0.01 for DDSM, and 0.68±0.02 for MIAS. The most important size features were related to the area covered by individual MC, eccentricity of individual MCs, eccentricity of MC cluster, MCs distances covered from MC cluster centroid, perimeter of MC cluster, and elongation of MC cluster.

We used 10-fold cross-validation with different seed values. The seed values initialize randomization of data in each fold. For example, if the value were set to 3, it would mean that the data were shuffled among the folds three times. Saving the seed value or setting it to the same number each time guarantees that the algorithm will come up with the same results—identical for each run. In this experiment, the seed number was set to 1 for the first run and its value was increased by 1 with each run. Hence, for 10 runs, the maximum seed value was set to 10. In 10-fold cross-validation, the original sample is randomly partitioned into 10 equal size sub samples (folds). Of the 10 sub samples, a single sub-sample is retained for testing the model, and the remaining (10 − 1) sub-samples were used as training data. The cross-validation process is then repeated 10 times, with each of the folds used exactly once as the test data. The 10 results from the folds are averaged to produce a single estimation. The advantage of this method is that all observations are used for both training and testing, and each observation is used for testing exactly once.

Note that the feature selection was only performed on the training data and therefore it was not expected that overfitting would happen. By using stratified 10-fold cross-validation, we avoided the risk of over-training. When using the ensemble learning and stack generalization approach, the hyper-parameters were kept as the default parameters set in Weka, since the advantage of using default parameters is that we eliminated the risk of introducing optimistic bias by tuning the parameter to maximize performance [53]. The segmentation and feature extraction were implemented using MATLAB Version 9.3.0.713579 (R2017b) on Windows 10. The features extracted from the images were converted from “.mat” format to “.arff” format to facilitate data structures as input for WEKA.

**Figure 10:**
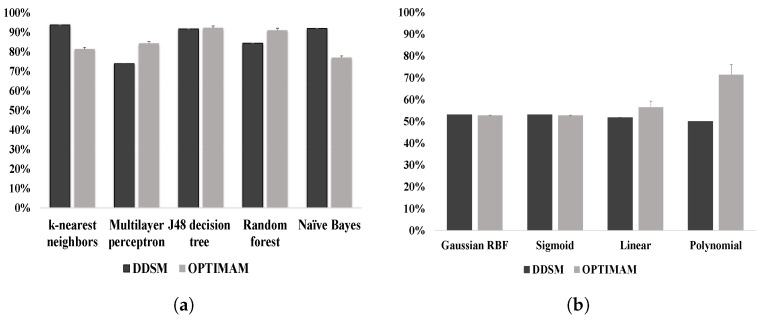
The accuracy of microcalcification cluster classification by individual classifiers: (**a**) classification accuracy for k-nearest neighbor, Multilayer perception, J48 decision tree, Random forests, and Naive bayes; and (**b**) classification accuracy by SVM using four different kernels: Gaussian RBF, Sigmoid, Linear, and Polynomial.

All nine classifiers, described in Section 4, were tested individually to assess their performance with results shown in Figure 10. SVM provided very low classification accuracy compared to the other classifiers, which is caused by low bias and high variance [54]. Another point to note is the SVM trained classifier used the trained data partly to estimate the margin, the support vectors, whereas others function classifiers considered the training set to define the decision function, making them more generalizable. When SVM was discarded from the classifier stack the overall classification performance decreased [11], while including SVM resulted in improved classification accuracy (around 90% for the DDSM database) [11], indicating the positive influence of SVM on ensemble learning, where a majority voting scheme was applied for improved generalization and to gain more flexibility to maintain strong prediction performance by averaging out classifiers individual mistakes and thus reducing the risk of over-fitting.

For the k-nearest neighbor (kNN) classifier, Figure 10a, the value of k was set to 5 based on cross-validation. The classification accuracy for digitized and digital mammograms was 93.77% and 81.37%, respectively. Lower value of k caused a decrease in classification accuracy and values higher than 5 provided the same accuracy as for k = 5. For a multilayer perceptron (MLP), the number of attributes were summed up with the number of classes and the result was divided by 2 to set the number of hidden layers whilst using the learning rate 0.3 and setting the validation threshold as 20 to terminate the validation testing. Such parameter settings were chosen because they provided the best classification accuracy for digital mammograms (around 84%), but the classifier showed poorer performance for digitized mammograms (around 73% classification accuracy). It is also worth noting that the accuracy increased to above 92% for both digital and digitized mammograms whilst using a classification tree, i.e., C4.5 (J48). Here, the confidence value was chosen to be 0.25 for pruning and the number of folds was set to 3, to determine the amount of data for reduced-error pruning and producing a decision tree. While applying a random forest, the accuracy for digitized mammograms was 84%, but the accuracy for digital mammograms was above 90%. The Naive Bayes classifier provided an increase in classification accuracy for digital mammograms (around 92%), but the accuracy decreased to around 76% for the digital database.

All classifiers were used to create an ensemble learner (see Section 4). The ensemble learner was applied to images from the three different databases: OMI-DB, DDSM, and MIAS. The performance of the ensemble learner is presented in Table 4. Ten-fold cross-validation (10-FCV) scheme and leave-one-out cross-validation (LOOCV) approach were used. For 10-FCV, the images were split into 10 folds, ensuring that each fold has the same proportion of observations with a given categorical value. In our experiment, each fold contained roughly the same proportions of the two types of class labels (benign and malignant). 10-FCV allows using different training and testing data, which avoids over fitting and gives better generalization ability. On the other hand, for LOOCV, each observation was held out with training based on the remaining samples.

**Figure 11:**
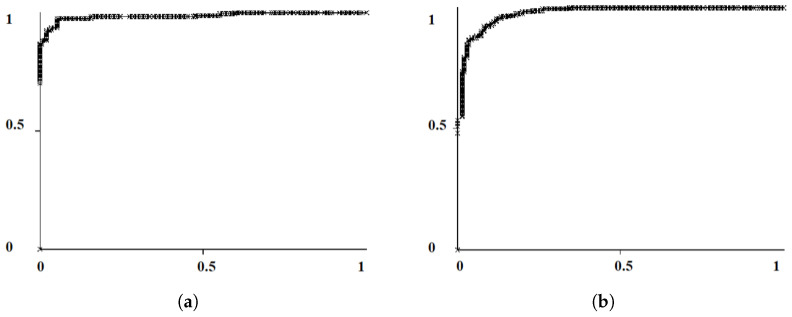
ROC curves for a stack generalization classifier for the OMI-DB digital database: (**a**) 2 features after feature selection (AUC = 0.97); and (**b**) 51 features (AUC = 0.96).

Two evaluation metrics were used. The first evaluation metric was the overall classification accuracy (CA), which was defined as the percentage of correctly classified MC clusters. The receiver operating characteristic (ROC) curve analysis was used as the second evaluation metric, plotting the true positive rate (TPR) against the false positive rate (FPR), which illustrated a whole range of possible operating characteristics for the classifier model. The ROC analysis was used to assess the predictive ability of the ensemble learner by using the area under the ROC curve denoted by A${}_{z}$ (also know as the AUC) [55] (see Figure 11). A${}_{z}$ is equivalent to the Wilcoxon signed-ranks test, which is a non-parametric alternative to the paired *t*-test [56]. All classification and evaluation aspects were implemented using the Weka [57] data mining suite.

When using 10-FCV, in Table 4, the ensemble learner performed better using only two important features, which were extracted and selected from the digital database (OMI-DB), showing an accuracy equal to 89.80±1.98%. The feature selection was performed using the proposed method described in Section 5. The two most important features were related to the MC cluster area and size of individual MC. Increase in accuracy was also noticed while using the same two important features to classify MC cluster for the digitized mammograms (85.24±2.52% for DDSM, and 100.00±0.00% for MIAS) compared to Table 3. When considering only the selected two important features, it was found that the classification accuracy achieved is lower for the digitized database (DDSM) than the accuracy achieved for the digital database (OMI-DB). A possible reason for such decrease in accuracy for the digitized mammograms is due to the decreased image quality compared to the digital mammograms, which affected the accuracy of the MC segmentation [58]. As the digital mammograms were higher quality, more accurate segmentation was obtained which potentially influenced appropriate feature extraction and classification results [59]. The accuracy was also high for the same selected features when using the LOOCV scheme: 91.12% for OMI-DB, 88.48% for DDSM, and 100% for MIAS. Such limitations of digitized mammograms were more pronounced when using four important features, extracted and selected from the digitized database (DDSM) using method explained in Section 5, and showed decreased accuracy when compared with the two selected features from the digital database (OMI-DB) (Table 4).

The stacked generalization approach [45] was applied to create an additional classifier, described in Section 4. The outputs of the nine different learning algorithms were collated to model a new dataset. The Naive Bayes classifier [44] was used as the meta-classifier to provide the final classification results [60]. The meta learner was used instead of averaging to combine the predictions of the base classifiers, which provided classification accuracy of 95.75% for the digital (OMI-DB) database, and classification accuracy of 95.17% for digitized database (DDSM) when applying only two important features that were extracted and selected from the digital database (OMI-DB) whilst using LOOCV scheme. With the same selected features, similar classification accuracy was obtained for OMI-DB (95.75±0.57%), and DDSM (94.90±0.72%) databases using 10-fold CV. As the precision for the digital (OMI-DB) and digitized (DDSM) databases are very similar; we performed an unpaired *t*-test, where *p* < 0.05 was obtained, indicating significant differences in the classification results using the digital and digitized databases. This demonstrates that our proposed classification approach works well, providing high classification accuracy for the digital databases (OMI-DB) over the digitized one (DDSM).

Comparing Table 4 and Table 5 signifies that the ensemble learner performs poorly, providing a decrease in the classification accuracy in all considered cases. This strongly supports the statement that the digital mammograms were higher quality, and more accurate segmentation was obtained which potentially regulate appropriate feature extraction and classification results [59]. It is worth noting that, even though 100% classification accuracy was obtained for the MIAS dataset, the number of sample in MIAS is very small (24 women, 12 benign and 12 malignant) to draw a significant conclusion in terms of classifying MC cluster, as it has smaller variability then the larger database like DDSM.

The results presented in Table 3, Table 4 and Table 5 are based on the images segmented maintaining the clinical grounding of the distribution of the MC cluster, which indicates that an area of 1 cm${}^{2}$ contains no fewer than three MCs [34]. Since the spatial resolution of mammography was 40–100 µm per pixel [15] which enabled the detection of MC clusters at an early stage —the feature extraction and selection method presented in Section 5 was employed on the segmented images from the digital and digitized databases that treated a 100×100 pixel block equivalent to a 1 cm${}^{2}$ area. This was done to investigate if such size selection had an impact on the MC cluster classification. The 100×100 pixel block is 50% of the block size (200×200) that was maintained to segment the digitized database (DDSM and MIAS) and 70% of the block size (143×143) that was maintained to segment the digital database (OMI-DB). In Table 6, both 51 features and the selected 4 most important features extracted from the digitized mammogram (DDSM) were used for MC cluster classification using LOOCV and 10-fold CV scheme. Here, the images were segmented, using the approach mentioned in Section 2.2.2, without following the clinical grounding of cluster distribution by selecting the block size 100×100 to investigate if it had any effects on the MC cluster classification.

The selected four most important features provided higher classification accuracy while applying LOOCV and 10-fold CV scheme for the OMI-DB database (95.77% for LOOCV, and 94.94±0.90% for 10-fold CV), the DDSM databases (93.91% for LOOCV, and 93.98±0.87% for 10-fold CV), and the MIAS database (100% for LOOCV, and 100.00±0.00% for 10-fold CV). Observing that the MIAS provided 100% classification accuracy with the four most important features, it had a very limited number of samples to draw significant conclusions. The increase in accuracy for the OMI-DB database with the four most important features over the 51 features derived from the digitized database (DDSM) warrant that the selected features from the digitized database (DDSM) have influence in classifying MC clusters in the digital mammograms (OMI-DB). This also demonstrated that the feature selection approach proposed in Section 5 is robust.

It is noteworthy that, whilst using 10-fold CV, the classification accuracy 94.94±0.90% for the OMI-DB database using the four most important features in Table 6, and the classification accuracy 95.75±0.57% for the same database using the two most important features in Table 5 appears to be similar. The same applied when comparing the classification accuracy for the DDSM database. With 10-fold CV and the four most important features (Table 6), the DDSM database achieved 93.98±0.87% classification accuracy, since with the two most important features and 10- fold CV, in Table 5, the DDSM database obtained an accuracy of 94.90±0.72%. The precision was calculated using an unpaired *t*-test for the aforementioned circumstances and *p* > 0.05 was obtained in all cases. This exhibits that similar classification accuracy can be achieved for classifying MC cluster using more number of features (four most important features) when the feature extraction and selection is performed on digitized database, whereas less features (two most important features) can be used to obtain similar classification accuracy (around 95%) when the feature extraction and selection is performed on digital database, and the MC are segmented complying with the clinical groundings concerning the cluster distribution.

## 7. Discussion

The proposed method for MC cluster classification was compared with other relevant publications (see Table 7). Akram et al. [12] proposed a tree-based representations for MC clusters, where scale-invariant topological features of MC were extracted showing 91% accuracy for cluster classification. Although high accuracy was achieved, the performance for MC cluster classification on digital mammogram was not reported in this study. In another study by Akram et al. [14], 96% classification accuracy was achieved using digitized mammograms with an improved Fisher Linear Discriminant Analysis (LDA) approach combined with a Support Vector Machine (SVM) variant.

The properties of MC clusters were presented by mereotopological barcodes by Strange et al. [58], where the discrete mereotopological relations between the individual MCs over a range of scales were presented in the form of a mereotopological barcode. The classification accuracy on digitized mammograms reported by Strange et al. [58] was 95% and 80% for the MIAS and DDSM datasets, respectively.

Chen et al. [15] used multi-scale graph topological features and classified MC clusters using k-nearest-neighbors-based classifiers. Their approach obtained 96% accuracy for digital mammograms. Though the accuracy for digital mammogram was high, the number of cases in the digital mammogram database was very low (25 cases), which provided less variability of MC distribution in the sample cases. It is also noteworthy that the digital images were manually annotated in Chen et al. [15], where delicate lines around small microcalcifications were outlined by an expert radiologist. Such delicate annotation with no false positives might result in higher classification accuracy. Chen et al. [15] also achieved high accuracy, around 95%, for a digitized database (MIAS) whilst again considering only a very small number of cases providing limited variation of MC clusters. Conversely, while using a large image database (DDSM), the classification accuracy reduced to 86% for a LOOCV approach and 85.20±0.05% for 10-fold CV. It is worth mentioning that only topological features were taken in to account to classify MC clusters, rather than concentrating on the morphological and statistical features of the MC clusters.

In our previous study [11], we acquire high classification accuracy (100%) for the MIAS database (24 cases) using LOOCV and 10-fold CV with an ensemble classifier. For DDSM, the accuracy was 91% (for LOOCV) and 90.02±1.42% (for 10-fold CV). The images used in Alam et al. [11] did not maintain the clinical grounding while segmenting the MC cluster using block processing approach. In addition, the experiment was not evaluated on digital mammograms. Promising results were achieved by our developed approach using the images from the digital and digitized databases (OMI-DB, DDSM, and MIAS). For brevity, we only show the results for the OMI-DB database in Table 7. The comparison of MC classification accuracy for the OMI-DB database with respect to the DDSM and MIAS databases is represented in Table 3, Table 4, Table 5 and Table 6 in Section 6. Whilst using an ensemble classifier for the OMI-DB database, 87.11±1.38% classification accuracy was achieved (see Table 4). For the DDSM database, the accuracy achieved was 76.28±1.25% for 10-fold CV, which was lower than for the OMI-DB database. The stack generalization approach, described in Section 4, was applied, which provided 89.85±1.69% accuracy without feature selection, and 95.75±0.57% accuracy with feature selection for the OMI-DB database (see Table 5). To perform quantitative evaluation for the stack generalization classifier, the receiver operating characteristic (ROC) curves for 2 features (Table 5) and 51 features are represented in Figure 11. Using ROC analysis, we achieved an area under the ROC of A${}_{z}$ = 0.97 when using 2 features, whereas for 51 features the value of A${}_{z}$ was 0.96. A${}_{z}$ is equivalent to the Wilcoxon signed-ranks test and a statistical measure, which is a non-parametric alternative to the paired *t*-test [61,62]. Additional details on feature selection are described in Section 5. A detailed discussion of the results can be found in Section 6.

In addition, Table 5 and Table 6 in Section 6 reveal that the stack generalization scheme outperformed the ensemble learning approach to classify MC clusters for both the digital and digitized mammograms using LOOCV and 10-fold CV approaches.

Apart from the classifiers that are described in Section 4, additional classification algorithms ([63,64,65]) were added to construct an extended ensemble learner which provided better classification accuracy 90.97±0.83% for the OMI-DB database (See Table 7) compared to the accuracy (87.11±1.38%) obtained by the ensemble learner initially used in Table 4 using 10-fold CV.

In Table 7, 95.95±0.57% accuracy was achieved with stack generalization with meta-classifier as Naive Bayes [44]. This accuracy was increased to 96.72±0.46% when using Adaptive boosting [66] as meta-classifier. The Adaptive boosting improved the performance accuracy as it produced a combined classifier whose variance is lower than the variances produced by the weak base learner [67].

It should be noted that most studies in Table 7 used smaller datasets, hence Table 7 represents a qualitative comparison. Table 7 shows how different classifiers classify MC clusters using different types of features. The methods were tested with different settings and data splitting. It is also important to note that the segmented images used in other classification approaches were based on the method proposed by Oliver et al. [38], whereas our proposed classification approach was based on the images segmented using the method proposed by Alam et al. [11], which is why the number of images from the same database in different experiments varied since the under-segmented images generated from the method proposed by Alam et al. [11] were discarded in our experiments. One significant drawback of the developed method was that it performed badly for cases where the MC clusters have no well-defined structure or very few MC were segmented in the cluster region. An extreme situation occurred when only a single MC was identified from the cluster by the segmentation approach explained in Section 2: this influenced the failure to discriminate malignant from benign based on individual MCs morphological feature and texture patterns. However, the experimental results demonstrated the robustness and effectiveness of the developed method when combined with automatic MC detection and feature selection.

## 8. Conclusions

We present a method for discriminating malignant and benign clusters in digital and digitized mammograms. Images from digital and digitized databases were first segmented using a wavelet based method incorporating bi-cubic interpolation and a series of morphological operations were carried out in order to facilitate the feature extraction and classification task from MC segmented images. A combination of morphological, texture, and distribution features from individual MC components and the whole MC clusters were extracted from mammograms. The most important features were selected and used to classify the MC cluster as benign or malignant. The clinical relevance of the selected features is discussed. ROC curve analysis was used to describe the cluster classification results. The feature extraction and selection were individually done using the digitized and digital mammograms, and afterwards those features were used to classify clusters in the digital database. The proposed method was evaluated using three different databases: OMI-DB, DDSM, and MIAS. Two different classifiers—ensemble learner and stack generalization—were applied to evaluate the classification result. The best classification accuracy (96.72±0.46%) for the digital database was achieved by using a stack generalization classification with 10-fold CV obtaining an A${}_{z}$ value equal to 0.98±0.00. 

## Figures and Tables

**Table 1:** Clinical description of the selected features using the DDSM database for classification of MC  clusters.

MC Cluster Classification Features	Radiologists Characterization Features
Summation of the mean of individual MC intensity	Density of MC cluster
Variance of the standard deviation of the distances from cluster centroids	MC distribution
MC cluster convex hull area	Cluster size
Mean of MC perimeter	Individual MC size

**Table 2:** Clinical description of the selected features using the OMI-DB database for classification of MC clusters.

MC Cluster Classification Features	Radiologists Characterization Features
MC cluster area	Cluster size
Size of individual MC	Individual MC size

**Table 3:** A${}_{z}$ estimation for the classification of MC clusters while applying 10-fold CV using ensemble learning on segmented image using a block size based on clinical rules.

Feature Selection	Feature Category	No. of Feature	Total Feature No.	A${}_{z}$ (AUC)		
				OMI-DB	DDSM	MIAS
	Size	17		0.87±0.01	0.75±0.01	0.74±0.03
No	Shape	17	51	0.70±0.02	0.69±0.02	0.61±0.04
	Texture	17		0.77±0.01	0.66±0.01	0.50±0.03
	Size	7		0.83±0.01	0.72±0.01	0.68±0.02
Yes	Shape	4	12	0.71±0.01	0.68±0.01	0.82±0.04
	Texture	5		0.78±0.02	0.68±0.01	0.67±0.03

**Table 4:** Classification accuracy using LOOCV and 10-fold CV applying all 51 and the 2 most salient features from digital mammogram, and 4 most salient features from the digitized mammogram using ensemble learning. The images were segmented following the clinical grounding of cluster distribution.

Database Name	Feature Number	LOOCV		10-FCV	
		CA	A${}_{z}$ (AUC)	CA	A${}_{z}$ (AUC)
OMI-DB	51	86.49%	0.85	87.11±1.38%	0.86±0.01
(286)	4	85.71%	0.84	83.55±2.57%	0.82±0.03
	2	91.12%	0.91	89.80±1.98%	0.89±0.02
DDSM	51	73.98%	0.73	76.28±1.25%	0.75±1.01
(280)	4	80.66%	0.80	81.67±1.65%	0.81±0.01
	2	88.48%	0.88	85.24±2.52%	0.82±0.08
MIAS	51	82.35%	0.79	95.29±4.41%	0.94±0.05
(24)	4	100.00%	1.00	100.00±0.00%	1.00±0.00
	2	100.00%	1.00	100.00±0.00%	1.00±0.00

**Table 5:** Classification accuracy using LOOCV and 10-fold CV applying all 51 and the 2 most salient features from digital mammogram, and 4 most salient features from the digitized mammogram using stacked generalization. The images were segmented following the clinical grounding of cluster distribution. Naive Bayes was used as the meta-classifier.

Database Name	Feature Number	LOOCV		10-FCV	
		CA	A${}_{z}$ (AUC)	CA	A${}_{z}$ (AUC)
OMI-DB	51	91.89%	0.97	89.85±1.69%	0.96±0.00
(286)	4	92.66%	0.98	92.70±0.63%	0.97±0.01
	2	95.75%	0.97	95.75±0.57%	0.97±0.01
DDSM	51	89.96%	0.95	89.74±1.35%	0.95±0.01
(280)	4	92.19%	0.96	93.12±0.58%	0.96±0.02
	2	95.17%	0.98	94.91±0.72%	0.97±0.01
MIAS	51	100%	1.00	97.06±2.94%	0.99±0.00
(24)	4	100%	1.00	100.00±0.00%	1.00±0.00
	2	100%	1.00	100.00±0.00%	1.00±0.00

**Table 6:** Classification accuracy using LOOCV and 10-fold CV applying all 51 and the 4 most salient features from digitized mammogram using stacked generalization. The images were segmented without following the clinical grounding of cluster distribution. Naive Bayes was used as the meta-classifier.

Database Name	Feature Number	LOOCV		10-FCV	
		CA	A${}_{z}$ (AUC)	CA	A${}_{z}$ (AUC)
OMI-DB	51	93.66%	0.97	91.38±0.86%	0.97±0.01
(286)	4	95.77%	0.98	94.94±0.90%	0.98±0.01
DDSM	51	90.68%	0.96	89.38±0.44%	0.94±0.01
(280)	4	93.91%	0.97	93.98±0.87%	0.96±0.02
MIAS	51	100%	1.00	99.58±1.25%	1.00±0.00
(24)	4	100%	1.00	100.00±0.00%	1.00±0.00

**Table 7:** A qualitative comparison of our results with respect to related work.

Method	Databases	Cases	Features	Classifier	Results
Akram et al. [12]	DDSM	288	Tree-based modeling	tree-structure height	CA = 91%
Akram et al. [14]	DDSM	288	Scalable−LDA	SVM	CA = 96%
Strange et al. [58]	DDSM	150	Cluster	barcodes	CA = 95%, A${}_{z}$ = 0.82
Strange et al. [58]	MIAS	20	Cluster	barcodes	CA = 80%, A${}_{z}$ = 0.80
Chen et al. [15]	MIAS I (Manual Annotation)	20	Topology	kNN/FNN/ FRNN/VQNN	CA = 95%, A${}_{z}$ = 0.96
Chen et al. [15]	Digital	25	Topology	kNN/FNN	CA = 96%, A${}_{z}$ = 0.96
Chen et al. [15]	DDSM (LOOCV)	300	Topology	kNN	CA = 86.0%, A${}_{z}$ = 0.90
Chen et al. [15]	DDSM (10-fold CV)	300	Topology	kNN	CA = 85.2±57%, A${}_{z}$ = 0.91±0.05
Alam et al. [11]	MIAS (LOOCV)	24	Morphology, Texture & Cluster	Ensemble classifier	CA = 100%, A${}_{z}$ = 1
Alam et al. [11]	MIAS (10-fold CV)	24	Morphology, Texture & Cluster	Ensemble classifier	CA = 100±0.00%, A${}_{z}$ = 1.00±0.00
Alam et al. [11]	DDSM (LOOCV)	280	Morphology, Texture & Cluster	Ensemble classifier	CA = 91.39%, A${}_{z}$ = 0.91
Alam et al. [11]	DDSM (10-fold CV)	280	Morphology, Texture & Cluster	Ensemble classifier	CA = 90.02±1.42%, A${}_{z}$ = 0.89±0.02
Ours	OMI-DB (10-fold CV)	286	Morphology, Texture & Cluster	Ensemble classifier (Extended)	CA = 90.97±0.83%, A${}_{z}$ = 0.91±0.01
Ours	OMI-DB (10-fold CV)	286	Morphology, Texture & Cluster	Stack generalization (meta-classifier: Naive Bayes)	CA = 89.84±1.69%, A${}_{z}$ = 0.96±0.00
Ours	OMI-DB (10-fold CV)	286	Morphology, Texture & Cluster (selected features)	Stack generalization (meta-classifier: Naive Bayes)	CA = 95.75±0.57%, A${}_{z}$ = 0.97±0.01
Ours	OMI-DB (10-fold CV)	286	Morphology, Texture & Cluster (selected features)	Stack generalization (meta-classifier: Adapting Boosting)	CA = 96.72±0.46%, A${}_{z}$ = 0.98±0.00
